# Effect of different concentrations of papain gel on orthodontic bracket bonding

**DOI:** 10.1186/2196-1042-14-22

**Published:** 2013-08-19

**Authors:** Matheus M Pithon, Caio S Ferraz, Gabriel D Couto Oliveira, Adrielle M Dos Santos

**Affiliations:** Department of Orthodontics, Southwest Bahia State University UESB, Av. Otavio Santos, 305 sala 705 Centro Odontomédico Dr. Altamirando da Costa Lima, Vitória da Conquista, Bahia, Brazil

**Keywords:** Shear strength, Composite resins, Orthodontic appliances, Papain

## Abstract

**Background:**

The objective of this study was to verify the hypothesis that enamel deproteinization with papain gel at concentrations of 2%, 4%, 6%, 8%, and 10% increases shear bond strength as concentration increases.

**Methods:**

A total of 180 bovine mandibular permanent incisors were used, divided into six groups (*n* = 30), and denominated as follows: group 1 is the control group (CG) in which brackets are bonded with resin-modified glass ionomer cement (RMGIC) according to the manufacturer's recommendations and groups 2, 3, 4, 5, and 6 have brackets bonded with RMGIC after enamel deproteinization with papain gel at concentrations of 2%, 4%, 6%, 8%, and 10%, respectively. After bonding, teeth were immersed in artificial saliva and kept at a temperature of 37°C for 24 h. Mechanical tests were then performed in a universal mechanical test machine EMIC DL 5000 (Sao Jose dos Pinhais, Brazil). Values obtained were submitted to analysis of variance and then to Tukey's test (*p* < 0.05).

**Results:**

The results demonstrated that groups 5 and 6 showed the highest shear bond strength, differing statistically from the other groups (*p* < 0.05). CG with no papain gel used showed the lowest value and in turn showed no differences for groups 2, 3, and 4. As regards adhesive remnant index, CG showed statistical differences from the others. Groups 2, 3, 4, 5, and 6, in which papain gel was used, presented no statistical differences among them (*p* > 0.05).

**Conclusions:**

It was concluded that enamel deproteinization with 8% and 10% papain gel increases shear bond strength of orthodontic brackets bonded with RMGIC.

## Background

In orthodontics, white stain lesions and marginal gingivitis are a source of concern to the majority of practitioners; oral hygiene measures and the use of new materials help to prevent these intercurrences [1–3]. Among these materials, glass ionomer cements (GICs) are outstanding as they allow chemical bonding to both enamel and dentin, in addition to releasing fluoride into the oral medium, accumulating it, and recharging themselves with it [4]. In spite of all these favorable characteristics, GICs do not provide adequate retention to enamel, resulting in a deficient bond between orthodontic accessories and enamel [5, 6].

Elimination of organic substances from the enamel surface before acid etching increases the resistance to orthodontic traction by providing a better acid etching pattern on enamel [7]. Deproteinization of the enamel surface before bracket bonding was first proposed by Justus [8]. This author used 5.25% sodium hypochlorite (NaOCl) for this purpose. NaOCl eliminates the organic matter present on the enamel surface by dissolving it [9]. Among the substances with similar activities, papain is outstanding.

Papain is extracted from the latex of *Carica papaya*, Caricaceae family, better known as papaya fruit. It is a cysteine enzymatic protein that shows antibacterial activity, has anti-inflammatory properties, and acts as an agent for debris removal, without any harmful effect on tissues because of the specificity of the enzyme [10, 11].

In 2003, papain was introduced into dentistry. The product, Papacárie®, (Formula e Ação, São Paulo, Brazil) is used in the chemical removal of caries, with the aim of removing infected tissue without causing any damage to any healthy structure in the mouth, using instruments without cutting edges and without the use of rotary instruments [10].

Recently, Pithon et al. [7] suggested the use of 10% papain as a deproteinizing agent before acid etching and verified that this removal of organic elements intensified the bond strength.

In the attempt to evaluate whether the effect of different concentrations of papain gel prior to orthodontic bracket bonding, the authors' proposal in the present study was to verify the hypothesis that with the increase in papain gel concentration, the shear bond strength values would be increased.

## Methods

A total of 180 bovine mandibular permanent incisors, extracted and stored in 10% formaldehyde solution, were used. After 7 days of fixation, they were cleaned, and the periodontal tissue that adhered to their roots was removed. When cleaning was concluded, they were fixed in reduction bushes measuring 25 × 20 (PVC, Tigre, Joinvile, Brazil) with self-polymerizing acrylic resin and stored in water under refrigeration for 7 days at a temperature of 5°C.

Initially, prophylaxis of all the teeth was performed with pumice stone (SSWhite, Juiz de Fora, Brazil) and water for 15 s, followed by washing for 15 s, and drying for an equal length of time. After this, the teeth were randomly divided into six groups (*n* = 15) that are denominated as follows:

1. Fixation with resin modified glass ionomer cement (RMGIC) according to the manufacturer's recommendations (etched with polyacrylic acid);
2. Deproteinization with 2% papain gel (Macela Dourada, Vitoria da Conquista, Bahia, Brazil), followed by fixation with RMGIC according to the manufacturer's recommendations;
3. Deproteinization with 4% papain gel (Macela Dourada, Vitoria da Conquista, Bahia, Brazil), followed by fixation with RMGIC according to the manufacturer's recommendations;
4. Deproteinization with 6% papain gel (Macela Dourada, Vitoria da Conquista, Bahia, Brazil), followed by fixation with RMGIC according to the manufacturer's recommendations;
5. Deproteinization with 8% papain gel (Macela Dourada, Vitoria da Conquista, Bahia, Brazil), followed by fixation with RMGIC according to the manufacturer's recommendations; and
6. Deproteinization with 10% papain gel (Macela Dourada, Vitoria da Conquista, Bahia, Brazil), followed by fixation with RMGIC according to the manufacturer's recommendations.

The orthodontic brackets (Morelli, Sorocaba, Brazil) for premolars were fixed in the center of the crown using RMGIC (3M Unitek, Monrovia, CA, USA). Excess adhesive and glass ionomer were removed from the teeth with a probe, and each bracket was then light-polymerized with a LED appliance (850 Mw/cm^2^) for 40 s (10 s on each side). After bonding, all samples were stored in artificial saliva (Apsen, São Paulo, Brazil) in a 37°C oven for 24 h.

A custom-made stand was used to stabilize the teeth for debonding tests. Each tooth was subjected to a shear load in a universal testing machine model DL-10.000 (EMIC, São José dos Pinhais, Brazil) with a knife-edged blade at a crosshead speed of 0.5 mm/min. The force was applied parallel to the tooth surface on top of each orthodontic bracket base, and the shear load was recorded at the point of failure. The force per unit area required to dislodge the bracket was then calculated and recorded as the shear bond strength in megapascals (MPa).

The enamel surfaces were examined with a stereomicroscope (Stemi 2000-C, Carl Zeiss, Gottingen, Germany) under ×16 magnification to determine the amount of composite remaining, and then they were classified according to adhesive remnant index (ARI). The ARI scores ranged from 0 to 3, with 0 indicating no composite left on the enamel; 1, less than half of the composite is left; 2, more than half of the composite is left; and 3, all of the composite remained on the tooth surface.

Statistical analyses were performed with the SPSS 13.0 program (SPSS Inc, Chicago, IL, USA). Descriptive statistics that included mean, standard deviation, median, and minimum and maximum values were calculated for all six groups. Analysis of variance was applied to determine whether significant differences existed among the groups. For the *post hoc* test, Tukey's test was used. Kruskal-Wallis and Mann-Whitney *U* tests were used for assessing ARI scores. The level of significance adopted was 5% (*p* = 0.05).

## Results and discussion

### Results

The results of the shear bond strength test demonstrated that the highest bond strength values were attained in the groups in which 8% and 10% papain were used, which differs statistically from the other groups (*p* < 0.05). The lowest values found were for the control group in which papain was not used. This in turn presented no statistical differences from the groups in which 2%, 4%, and 6% papain was used (Table 1).

**Table 1:** **Minimum, maximum, mean, and SD of shear bond strength values and statistical analysis of evaluated groups**

Groups	Minimum	Maximum	Mean (SD)	Significance	Statistics
(1) Control	0.2	16.6	3.3 (2.4)	(2) *p* = 0.277	A
				(3) *p* = 0.126	
				(4) *p* = 0.117	
				(5) *p* = 0.019	
				(6) *p* = 0.017	
(2) Papain 2%	2	11.2	5.7 (2.4)	(3) *p* = 0.999	A
				(4) *p* = 0.998	
				(5) *p* = 0.867	
				(6) *p* = 0.852	
(3) Papain 4%	2.1	12.3	6.1 (3.5)	(4) *p* = 1.00	A
				(5) *p* = 0.976	
				(6) *p* = 0.972	
(4) Papain 6%	0.2	16.3	6.1 (3.9)	(5) *p* = 0.981	A
				(6) *p* = 0.977	
(5) Papain 8%	2.3	15.7	6.9 (3.6)	(6) *p* = 1.00	B
(6) Papain 10%	3.4	10.3	7.0 (1.6)		B

*SD*, Standard deviation. Equal letters correspond to the absence of statistical differences (*p* < 0.05).

As regards the ARI, the control group showed statistical differences from the others, as can be observed in Table 2. The other groups, in which papain was used, presented no statistical differences among them.

**Table 2:** **Mean ARI values of the evaluated groups**

Groups	25th percentile	75th percentile	Median	Significance
(1) Control	0	1	1	(2) *p* = 0.004
				(3) *p* = 0.004
				(4) *p* = 0.001
				(5) *p* = 0.001
				(6) *p* = 0.001
(2) Papain 2%	1	2	2	(3) *p* = 0.744
				(4) *p* = 0.367
				(5) *p* = 0.412
				(6) *p* = 0.567
(3) Papain 4%	1	2	2	(4) *p* = 0.624
				(5) *p* = 0.653
				(6) *p* =0.902
(4) Papain 6%	2	2	2	(5) *p* = 1.00
				(6) *p* = 0.683
(5) Papain 8%	1.5	2	2	
(6) Papain 10%	1.5	2	2	

*SD*, Standard deviation.

## Discussion

Decalcification is an important effect of orthodontic therapy on tooth enamel as orthodontic accessories and their bonding materials create retentive areas around them for bacterial biofilm accumulation [12]. This allied to poor oral hygiene habits and leads to the formation of white stain lesions and marginal gingivitis adjacent to fixed orthodontic appliances [1, 13].

With the intention of minimizing and preventing white spot lesions, there is awareness about the use of new fluoride-releasing materials [6].

Glass ionomer cements were developed with the aim of uniting biological and chemical properties in one and the same material. In addition to promoting fixation to the tooth surface, they release and are recharged with fluoride, thereby reducing white stain lesions around brackets [6, 14–16].

However, the GICs have lower bond strength to the enamel surface in comparison with orthodontic composites. With the intention of associating important characteristics of the two materials, such as shear bond strength and fluoride release, resin modified glass ionomer cements were developed, which release fluoride without compromising the bond strength to the tooth surface [6].

The bond strength between orthodontic accessories and enamel may be compromised by the presence of the acquired pellicle at the time when they are being bonded [17–20]. It is well known because it is a biologically important integument on the tooth surface as it constitutes the interface between the enamel surface and the first layer of oral biofilm. It is recognized that at a functional level, it plays a role in mineral homeostasis of tooth enamel. There is ample evidence that this structure is formed by selective adsorption of proteins, peptides, and other molecules present in oral fluid [18, 20]. Therefore, it is of fundamental importance to remove the acquired pellicle by deproteinizing it before performing orthodontic bracket bonding.

With the purpose of eliminating the influence of the organic matrix on the adhesion of composites to the enamel surface, Justus [8] suggested the use of 5.25% NaOCl for 60 s, and Pithon suggested the use of 10% papain for 60 s as deproteinizing agent before etching with 37% phosphoric acid, showing good results.

The aim of the present study was to verify the hypothesis that deproteinization of the enamel surface with 2%, 4%, 6%, 8%, and 10% papain gel for 60 s increases the shear bond strength of brackets bonded with RMGIC.

Deproteinization performed with papain gel removes the acquired pellicle, which persists after prophylaxis, from the tooth surface [7].

The gel at concentrations of 2%, 4%, 6%, 8%, and 10% is obtained from an alkaloid enzyme, papain, extracted from the papaya fruit (*Carica papaya*). It is extracted from the latex in the leaves and skin of the mature fruit. In addition to the proteolytic action, it has antibacterial and anti-inflammatory properties, thus acts as remover of necrotic remainders, and is not cytotoxic [11, 21, 22].

Papacárie® is papain presented in gel form and differs from that used in the present study. It is not formulated from pure papain but is made up of chloramine - a compound of chlorine and ammonia used for root canal irrigation and toluidine blue dye - photosensitizer with antimicrobial properties, and papain [22]. In this study, the gel was used because in this presentation, it is easy to manipulate. In this study, it was perceived that in concentrations of up to 6%, in groups 2, 3, and 4, papain gel showed no statistical differences from the control group, and its use in these concentrations is not clinically relevant, considering that its use adds a clinical step without any real gain in bond strength.

Deproteinization is a prudent step to include when using RMGIC as there is an improvement in marginal sealing at the base of the accessory with the enamel surface, in addition to the formation of white stain lesions being minimized with the use of RMGIC [8].

The enamel surface etched with 37% phosphoric acid after eliminating the organic elements from it probably produces more tags that penetrate into the enamel. These lead to a significant increase in mechanical retention of adhesives to enamel, particularly RMGICs, as demonstrated in the present study [8].

The use of papain as deproteinizing agent increases the shear bond strength irrespective of the etching agent [7].

When comparing the mean shear bond strength values presented by the groups (3, 4, 5, and 6), with the values suggested by Reynolds [22], these are adequate for the majority of procedures performed in orthodontics (between 5.9 and 7.8 MPa).

Apart from the groups already mentioned, papain gel was also tested at concentrations of 8% and 10%. These obtained better results in comparison with the other groups and presented no statistical differences between them. Thus, the concentrations of 8% and 10% increased the RMGIC bond to the enamel surface, adding another favorable characteristic to this material when one thinks of using it for bonding orthodontic accessories, a result that corroborates Pithon's findings [7].

As regards the ARI, the control group presented statistical differences from the other groups (2, 3, 4, 5, and 6), and these showed no statistical differences among them. This result is clinically relevant considering that papain gel at all tested concentrations increased the RMGIC bond to tooth enamel and also avoided damage to enamel when the accessories were debonded.

The results presented here are given preliminaries that were coming from an *in vitro* study. *In vivo* studies should be performed in order to confirm our findings.

The results presented here are preliminaries given that were coming from an in vitro study. In vivo studies should be performed in order to confirm our findings.

## Conclusion

By conducting this study, the following conclusions were drawn:

- The formulated hypothesis was proven since the papain gel at concentrations of 8% and 10% significantly increased the shear bond strength of RMGIC.
- As regards the ARI, papain gel at concentrations of 2%, 4%, 6%, 8%, and 10% increased the RMGIC bond to the enamel surface, which is clinically relevant, as they minimize the occurrence of cracks and fractures on the tooth surface when orthodontic accessories are removed.

## Authors’ information

MMP is a professor in Orthodontics at Southwest Bahia State University (UESB). He is a diplomate of the Brazilian Board of Orthodontics (BBO) and is a specialist in Orthodontics at the University of Alfenas - Unifal. He received his MSc and PhD in Orthodontics from Federal University of Rio de Janeiro (UFRJ). CSF, GCO, and AMS are Dentistry students of Southwest Bahia State University (UESB).
